# Supplementary material for: Optimization of differentiation and transcriptomic profile of THP-1 cells into macrophage by PMA
Source: PLoS One. 2023 Jul 17;18(7):e0286056. doi: 10.1371/journal.pone.0286056 (PMC10351730; doi:10.1371/journal.pone.0286056)
Supplement: S1 File — (ZIP) [file pone.0286056.s001.zip › supporting files/RNA-seq report.zip!/RNA-seq report/2_GeneQuantification_result/cor_pearson.pdf]

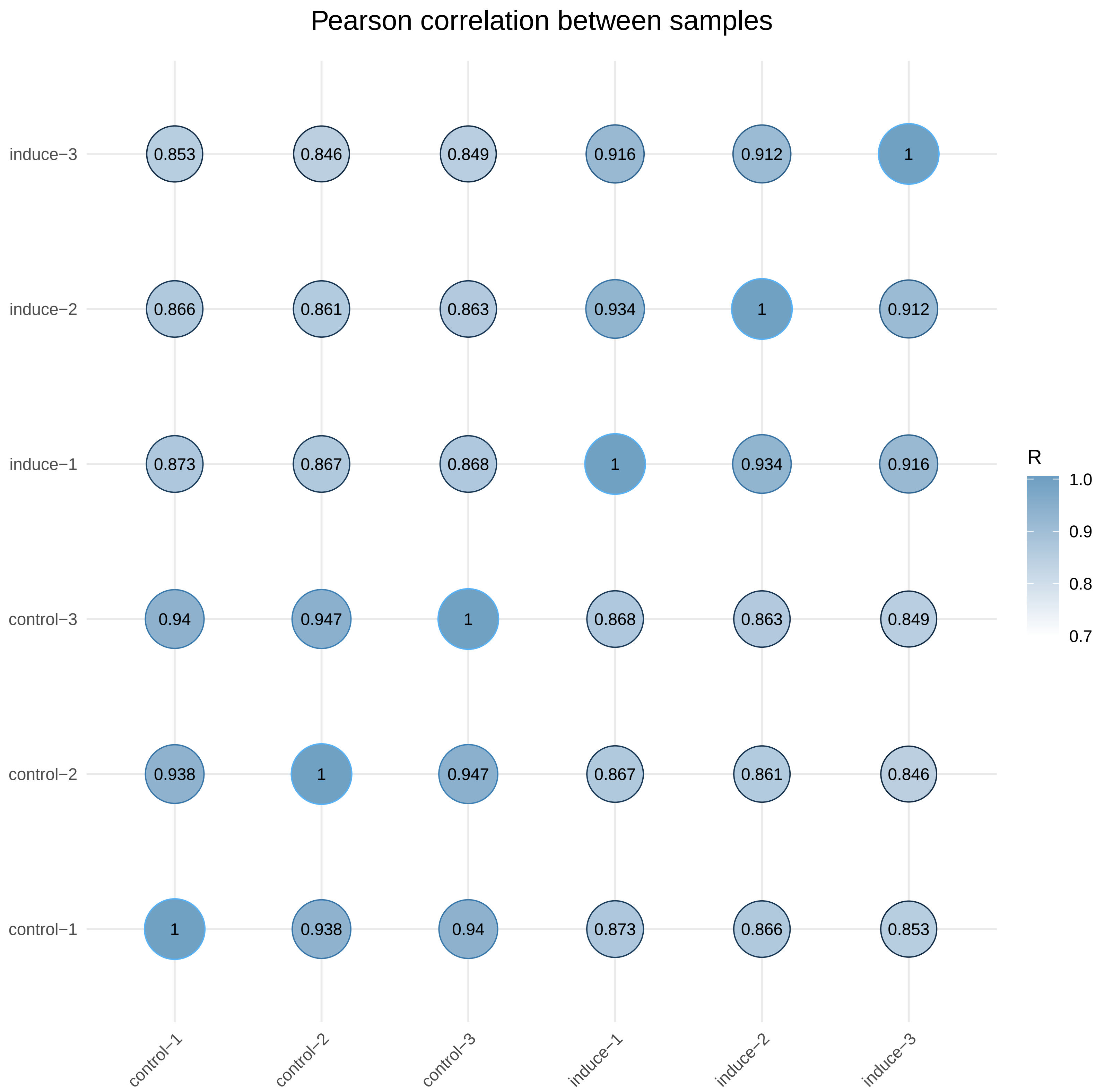
Pearson correlation between samples
induce−3
induce−2
induce−1
control−3
control−2
control−1
control−1
control−2
control−3
induce−1
induce−2
induce−3
0.853
0.846
0.849
0.916
0.912
1
0.866
0.861
0.863
0.934
1
0.912
0.873
0.867
0.868
1
0.934
0.916
0.94
0.947
1
0.868
0.863
0.849
0.938
1
0.947
0.867
0.861
0.846
1
0.938
0.94
0.873
0.866
0.853
R
1.0
0.9
0.8
0.7
